# MUC1-associated proliferation signature predicts outcomes in lung adenocarcinoma patients

**DOI:** 10.1186/1755-8794-3-16

**Published:** 2010-05-06

**Authors:** Dhara M MacDermed, Nikolai N Khodarev, Sean P Pitroda, Darrin C Edwards, Charles A Pelizzari, Lei Huang, Donald W Kufe, Ralph R Weichselbaum

**Affiliations:** 1 The Scripps Research Institute and Scripps Translational Science Institute, 3344 N. Torrey Pines Court Ste. 300, La Jolla, CA, 92037, USA; 2 Department of Radiation and Cellular Oncology, The University of Chicago, Duchossois Center for Advanced Medicine, 5758 S. Maryland Avenue, MC9006, Chicago, IL 60637, USA; 3 Department of Radiology, The University of Chicago Medical Center. 5841 S. Maryland Ave., Chicago, IL 60637, USA; 4 Dana-Farber Cancer Institute, Harvard Medical School, 44 Binney St., Dana 830, Boston, MA 02115, USA

## Abstract

**Background:**

MUC1 protein is highly expressed in lung cancer. The cytoplasmic domain of MUC1 (MUC1-CD) induces tumorigenesis and resistance to DNA-damaging agents. We characterized MUC1-CD-induced transcriptional changes and examined their significance in lung cancer patients.

**Methods:**

Using DNA microarrays, we identified 254 genes that were differentially expressed in cell lines transformed by MUC1-CD compared to control cell lines. We then examined expression of these genes in 441 lung adenocarcinomas from a publicly available database. We employed statistical analyses independent of clinical outcomes, including hierarchical clustering, Student's t-tests and receiver operating characteristic (ROC) analysis, to select a seven-gene MUC1-associated proliferation signature (MAPS). We demonstrated the prognostic value of MAPS in this database using Kaplan-Meier survival analysis, log-rank tests and Cox models. The MAPS was further validated for prognostic significance in 84 lung adenocarcinoma patients from an independent database.

**Results:**

MAPS genes were found to be associated with proliferation and cell cycle regulation and included CCNB1, CDC2, CDC20, CDKN3, MAD2L1, PRC1 and RRM2. MAPS expressors (MAPS+) had inferior survival compared to non-expressors (MAPS-). In the initial data set, 5-year survival was 65% (MAPS-) vs. 45% (MAPS+, p < 0.0001). Similarly, in the validation data set, 5-year survival was 57% (MAPS-) vs. 28% (MAPS+, p = 0.005).

**Conclusions:**

The MAPS signature, comprised of MUC1-CD-dependent genes involved in the control of cell cycle and proliferation, is associated with poor outcomes in patients with adenocarcinoma of the lung. These data provide potential new prognostic biomarkers and treatment targets for lung adenocarcinoma.

## Background

Lung cancer is the most common cancer worldwide and is the leading cause of cancer-related death in the United States. Approximately 213 000 new diagnoses and over 160 000 deaths from lung cancer occur annually in the United States [1]. About 85% of lung cancers are non-small cell histology (NSCLC), including lung adenocarcinoma, which is the most common lung cancer type [2]. Treatment of early and intermediate stage NSCLC usually involves surgery. Most patients with localized lung cancer are now treated with adjuvant platinum-based chemotherapy, which provides a survival advantage [3]. The utility of postoperative radiation is controversial and subsets of patients have been proposed to benefit, but clear clinical and/or molecular identification of patients who may benefit from postoperative radiation remains uncharacterized. In contrast, recently identified molecular classifiers based on statistically derived gene signatures may facilitate the selection of patients who will benefit from adjuvant chemotherapy [4,5]. Nonetheless, no prognostic or predictive signature for NSCLC is regularly used in a clinical setting.

Mucin 1 (MUC1) is a protein heterodimer that is overexpressed in lung cancers [6]. MUC1 consists of two subunits, an N-terminal extracellular subunit (MUC1-N) and a C-terminal transmembrane subunit (MUC1-C). Overexpression of MUC1 is sufficient for the induction of anchorage independent growth and tumorigenicity [7]. Other studies have shown that the MUC1-C cytoplasmic domain is responsible for the induction of the malignant phenotype and that MUC1-N is dispensable for transformation [8]. Overexpression of MUC1 also confers resistance to stress-induced cell death, conferred by exposure to certain genotoxic anticancer agents [9-11]. In this regard, targeting of the MUC1-CD subunit to the nucleus attenuates p53-mediated apoptosis in response to DNA damage [12]. Notably, MUC1 protein expression has been associated with poor prognosis in NSCLC [13,14]. Taken together, these data have provided a rationale for an in-depth analysis of transcriptional programs induced by the MUC1-C cytoplasmic domain (MUC1-CD).

We previously reported a method for analysis of biologically derived data relevant to the identification of expressional signatures with prognostic and predictive value [15-17]. We used this approach to identify a MUC1-induced Tumorigenesis Signature (MTS) based on the profiling of MUC1-CD-transfected xenografts grown in nude mice [18]. The MTS was derived through comparison of MUC1-CD-transfected tumors *in vivo *and the corresponding cell lines grown *in vitro*. We hypothesized that such a comparison would detect the genes differentially expressed as a result of tumor-stromal interactions. Indeed, the major functional groups of genes represented in MTS were cell motility, metastasis and angiogenesis. In the current report, we focused on the *in vitro *profiling of 3Y1 cell lines transfected with MUC1-CD compared with mock-transfected cells to define "intrinsic" MUC1-CD-dependent transcriptional changes without stromal effects. Using this approach, we expected to identify MUC1-CD-dependent genes intrinsically associated with an aggressive tumor behavior. Here we report that a MUC1-Associated Proliferation Signature (MAPS) comprised of genes that mediate cell cycle control and mitotic spindle assembly has significant prognostic value in lung adenocarcinoma patients. Importantly, the MAPS is the first biologically derived gene signature comprised uniquely of MUC1-induced genes involved in the control of cell cycle and proliferation.

## Methods

### Cells and culture conditions

Rat 3Y1 embryonic fibroblasts were transfected by an empty vector (3Y1/Vector) and by the cytoplasmic domain of MUC1 (3Y1/MUC1-CD) as previously described [19]. Transfected 3Y1 cells were cultured in DMEM media with 10% heat-inactivated fetal bovine serum, 100 units/mL penicillin, 100 μg/mL streptomycin, and 2 mmol/L L-glutamine and maintained at 37°C in a humidified environment containing 5% CO_2_.

### RNA extraction and purification

RNA was collected and purified from confluent 3Y1/Vector and 3Y1/MUC1-CD cell cultures using TRIzol reagent (Invitrogen Life Sciences, Carlsbad, CA, USA) according to the manufacturer's instructions. Further purification was performed using a combination of RNeasy spin columns and TRIzol reagent, as we previously described [20]. The quality of samples was assessed using gel electrophoresis in 1.8% agarose and spectrophotometry, and samples of high quality were transferred to the Functional Genomics Facility of The University of Chicago for labeling and hybridization with GeneChip^® ^Rat Genome 230 2.0 Arrays (Affymetrix, Santa Clara, CA, USA).

### DNA microarray data collection and analysis

The selection and analysis of genes differentially expressed in 3Y1/Vector cells compared to 3Y1/MUC1-CD cells *in vitro *was based on previously described methods. Briefly, each array was hybridized with a pooled sample normalized to total RNA and consisting of RNA obtained from 3 independent cell lines. After data retrieval and scaling using MAS 5.0 Microarray Suite software (Affymetrix, Santa Clara, CA, USA), data were rescaled using "global median normalization" across the entire dataset [21] and filtrated using a multi-step filtration method, which involves the application of Receiver Operating Characteristic analysis (ROC analysis) for the estimation of cut-off signal intensity values [22]. Subsequent analysis was based on pair-wise comparisons of duplicated arrays (3Y1/Vector *in vitro *vs. 3Y1/MUC1-CD *in vitro*) using Significance Analysis of Microarrays (SAM) version 3.0 (Stanford University Labs, http://www-stat.stanford.edu/~tibs/SAM/). Differentially expressed probe set IDs were selected using a 2.0-fold induction cut-off level with selection of delta values minimizing the False Discovery Ratio. Probe set IDs were gene annotated and functionally designated using Ingenuity Pathways Analysis (IPA, Ingenuity^® ^Systems, http://www.ingenuity.com).

Array data are deposited in GEO, accession # GSE14337 (MUC1-induced transcriptional alterations in rat 3Y1 embryonic fibroblasts [Rattus norvegicus]), http://www.ncbi.nlm.nih.gov/geo/query/acc.cgi?acc=GSE14337.

### Collection of publicly available cancer databases

Two publicly available cancer databases containing expressional data from adenocarcinoma of the lung were analyzed to determine whether the MUC1-CD-dependent genes have predictive value in determining the outcome for each patient sample. The first database was obtained from a multicenter consortium (University of Michigan Cancer Center, Moffitt Cancer Center, Memorial Sloan-Kettering Cancer Center, and the Dana-Farber Cancer Institute) consisting of surgically resected lung adenocarcinoma specimens from 442 patients [23]. These patients presented with stage I to III disease and were treated without pre-operative chemotherapy or radiation. A subset of patients (n = 108) had adjuvant treatment with radiation and/or chemotherapy. One patient was excluded from survival analysis because survival data was missing. An independent database was used for confirmation of our findings [24]. Analysis included 84 quality-controlled cases with at least 40% tumor cellularity, adenocarcinoma only (no mixed histology), and available survival data [25]. These patients were treated with primary surgical resection and data regarding adjuvant therapy were not available.

### Statistical analyses

For initial analysis, the raw signal intensity for each probe set ID of interest for each patient was normalized to the median value of the probe set ID across the entire database and subsequently log_2_-transformed. For subgroup analyses, the raw data were divided into subgroups and normalization was performed across each subgroup. Multiple probe set IDs for a given gene were averaged for each patient sample to obtain a representative expression value for each gene.

Clustering and survival analyses were performed using JMP 7.1 (SAS Institute Inc. Cary, NC, USA). Expression data were clustered using hierarchical clustering via Ward's method to visualize gene expression patterns across each database. Genes having uniform expression across the patient samples in the initial clustering of genes were eliminated and not used for further analyses (87 out of 254 genes were eliminated in this manner). Before any further reduction of the gene set, survival analysis was performed based on clustering of the biologically derived genes. To determine whether clustering based on the differential expression of the MUC1-CD-induced genes could identify patients with decreased survival, Kaplan-Meier survival analysis was performed on clusters defined by k-means clustering. The k-means clustering was performed using a predetermined number of clusters (k = 2) and a log-rank test was used to estimate significance of survival differences between the two clusters. Subsequently, a smaller number of genes was selected from this gene set for practical application. An F-test was further used to test the null hypothesis of no difference in variance for each gene between the two patient clusters. Results of the F-test were further entered into an unpaired 2-tailed Student's t-test to test the null hypothesis of no difference in the magnitude of gene expression of each gene between the two patient clusters. The alpha level for each t-test was 0.05. As a result, we obtained a set of probes comprising 42 unique genes. Once the final signature was selected using ROC analysis (see full details in subsequent ROC analysis section), the mean expression across genes in the signature was calculated as a relative expression score [17,18]. A positive value (> 0) designated the patient as MAPS+. Kaplan-Meier survival statistics were performed and log-rank tests were used to test the null hypothesis of no difference in survival functions between MAPS+ and MAPS- patients. We then performed univariate and multivariate analysis using a Cox proportional hazards model to evaluate prognostic factors, including MAPS. Clinical features such as tumor stage, lymph node involvement and histological grade were included in these analyses as binary variables: T1-T2 vs. T3-T4, nodes involved (N1-2) vs. uninvolved (N0), and intermediate or high grade vs. low grade. For each clinical variable, binary categories were selected based on maximum prognostic significance for overall survival in our dataset.

### Receiver operating characteristic (ROC) analysis

To derive the final 7-gene signature, ROC analysis was used to assign an AUC value to each gene as a single feature. A ROC curve is a graphical plot of the sensitivity vs. (1 - specificity) for a binary classifier system as its discrimination threshold is varied. The area under the ROC curve (AUC) is a widely-used performance metric for the binary classifier system being evaluated. We used ROC analysis to assign area under the curve (AUC) scores to evaluate the ability of each individual gene in the set to classify patients into the two groups (expressors vs. non-expressors - see [26]). This analysis was performed independent of survival data. AUC scores were assigned to each gene by applying the PROPROC program [27]. We selected those genes with an AUC score above 0.95, representing a probability of error under 5%, which resulted in a subset of 7 genes. The algorithm by which we narrowed our gene set down to 7 genes is summarized in Additional File 1, Figure S1. Six of the seven genes had matching expressional data in the validation dataset (PRC1 was absent in this database due to the differences in array platforms).

## Results

### 1. MUC1 is associated with changes in the expression of genes that regulate cellular growth and proliferation

**Figure 1:**
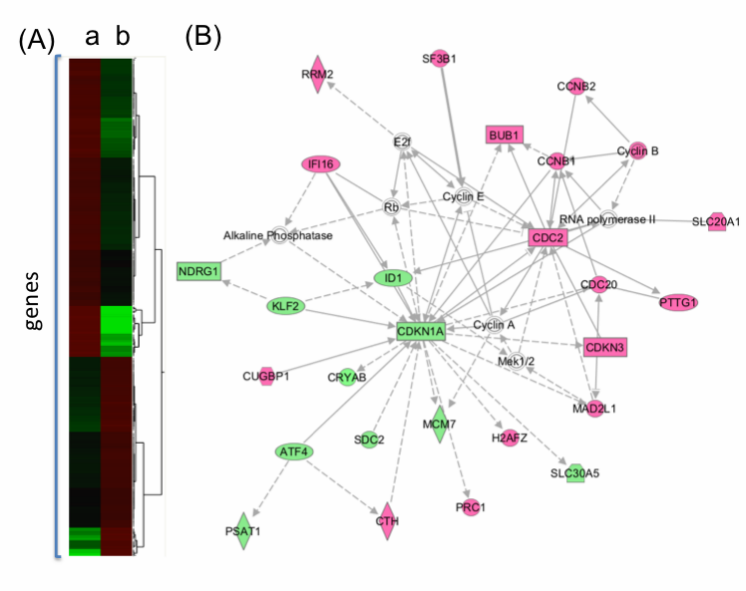
**Differentially expressed genes in MUC1 transfected cells**. **A**. Expressional clustering of genes expressed at least two-fold differently in (a) MUC1 transfected 3Y1 cells compared with (b) cells transfected with empty vector. **B**. A functional network involving many of these upregulated (pink) and downregulated (green) genes. Functions include cell cycle, cellular assembly and organization, DNA replication, recombination and repair.

Differences in gene expression were identified by comparing MUC1-CD-transformed 3Y1 cells to those transfected with a control vector and grown *in vitro*. Gene expression analysis using Significance Analysis of Microarrays (SAM) yielded 254 differentially expressed genes in MUC1-CD-transfected cells, shown in Figure 1A and Additional File 2, Table S1. Functional gene analysis using Ingenuity Pathway Analysis (IPA) of the 254 genes identified cellular growth and proliferation as the most significantly represented function (91 molecules, Fisher's exact *p *= 1.26 × 10^-8^). Furthermore, IPA functional network analysis demonstrated that the most statistically significant networks representing the web of interactions among these genes indeed mediate cellular growth and proliferation (Fisher's exact *p *= 10^-44^) and cell cycle and cellular assembly (Fisher's exact *p *= 10^-41^) as shown in Figure 1B and Additional File 3, Table S2. Taken together, these data indicate that MUC1-CD-induced transformation is associated with distinct changes in gene expression associated with the control of cellular growth and proliferation.

### 2. Development of a MUC1-dependent, proliferation-associated molecular signature

**Figure 2:**
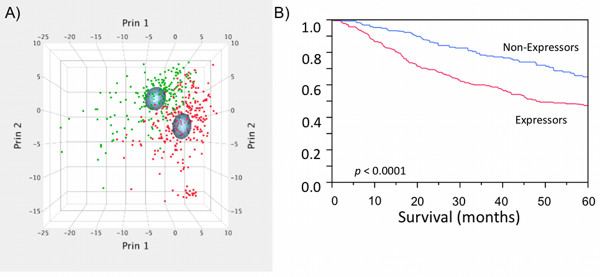
**Expressional clustering based on 254 MUC1-CD-associated genes is associated with survival of lung adenocarcinoma patients**. **A**. 3-dimensional representation of the centroids generated by K-means clustering. Each point forming a cloud surrounding the centroid represents a patient assigned to the cluster corresponding to the centroid. **B**. 5-year survival of patients assigned to each cluster.

To test the hypothesis that differential expression of the 254 genes regulated by MUC1-CD is linked to poor prognosis, a large, multi-institutional lung adenocarcinoma database of 442 cases [23] was utilized. K-means clustering based on expression of these 254 genes was used to divide patients into two groups (Figure 2A). Kaplan-Meier survival analysis of patient groups defined by k-means clustering demonstrated significant 5-year survival differences between groups (47.0% vs. 64.6%, log-rank p < 0.0001, Figure 2B). These data demonstrate that differential expression of MUC1-CD-associated genes is associated with poor outcome of lung adenocarcinoma patients.

**Figure 3:**
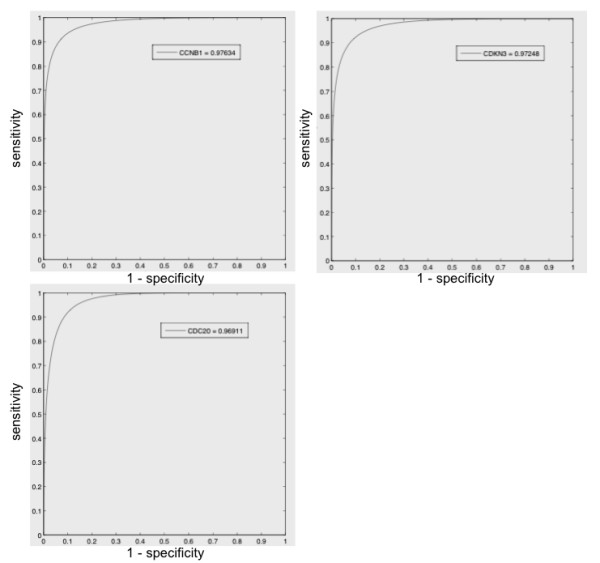
**ROC curves analyzing the use of expression levels of individual genes, CCNB1, CDC20 and CDKN3, to accurately assign patients to prognostic groups**.

We used a combination of parametric statistics and receiver operating characteristic (ROC) analysis to reduce the size of this gene set and identify those genes whose expression was most closely linked to the prognostic clusters identified using the entire 254 gene set. Forty-two genes were initially identified using parametric statistics (see Table 1). Network analysis using IPA showed that these genes form a network representing DNA replication/cell cycle, recombination and repair (e.g. CCNB1, CDC2, CDC20, CDKN3, MAD2L1 and MCM7). For more details on this network, see Additional File 4, Figure S2. Similar data were obtained by IPA-based analysis of the most significant functions of these 42 genes, which represented the same pathways (*p *= 1.5 × 10^-6^; Fisher's exact test). Seven genes, including CCNB1, CDC20, CDKN3, CDC2, MAD2L1, PRC1, and RRM2, were identified through ROC analysis (see Table 2). Representative ROC curves for the top three genes (CCNB1, CDC20 and CDKN3) are shown in Figure 3. Given that IPA analysis of these genes demonstrated significant functions in the regulation of cell proliferation, cell cycle and chromosome segregation, we designated this 7-gene set as the MUC1-Associated Proliferation Signature (MAPS).

**Table 1:** 42 genes selected based on their differential expression between prognostic groups.

Gene Symbol	Gene Title
ALDH6A1	aldehyde dehydrogenase 6 family

BECN1	beclin 1

BTG3	BTG family

BUB1	BUB1 budding uninhibited by benzimidazoles 1 homolog (yeast)

CCNB1	cyclin B1

CDC2	cell division cycle 2

CDC20	cell division cycle 20 homolog (S. cerevisiae)

CDKN3	cyclin-dependent kinase inhibitor 3

CKAP4	cytoskeleton-associated protein 4

CSRP2	cysteine and glycine-rich protein 2

DEPDC1	DEP domain containing 1

DHCR24	24-dehydrocholesterol reductase

DNAJC9	DnaJ (Hsp40) homolog

DUSP6	dual specificity phosphatase 6

EDEM1	ER degradation enhancer

EHD4	EH-domain containing 4

ELOVL6	ELOVL family member 6

ERN1	endoplasmic reticulum to nucleus signaling 1

ETV4	ets variant gene 4 (E1A enhancer binding protein

FRS2	fibroblast growth factor receptor substrate 2

FUCA1	fucosidase

H2AFZ	H2A histone family

ID1	inhibitor of DNA binding 1

KAZALD1	Kazal-type serine peptidase inhibitor domain 1

LRRC17	leucine rich repeat containing 17

MAD2L1	MAD2 mitotic arrest deficient-like 1 (yeast)

MCM7	minichromosome maintenance complex component 7

OLFML3	olfactomedin-like 3

PFN2	profilin 2

PGCP	plasma glutamate carboxypeptidase

PJA2	praja 2

PRC1	protein regulator of cytokinesis 1

PTPRF	protein tyrosine phosphatase

RRM2	ribonucleotide reductase M2 polypeptide

SC5DL	sterol-C5-desaturase (ERG3 delta-5-desaturase homolog

SDC2	syndecan 2

SLC16A1	solute carrier family 16

SLC20A1	solute carrier family 20 (phosphate transporter)

SSX2IP	synovial sarcoma

TRIM25	tripartite motif-containing 25

TXNDC13	thioredoxin domain containing 13

**Table 2:** Top scoring genes from ROC analysis.

Gene Symbol	Gene Title	Pathway	AUC score
CDC20	Cell division cycle 20	Cell cycle	0.99

RRM2	Ribonucleotide reductase M2 polypeptide	DNA synthesis	0.98

CCNB1	cyclin B1	Cell cycle G1 to S	0.98

MAD2L1	MAD2 mitotic arrest deficient-like 1 (yeast)	Mitotic spindle checkpoint	0.98

PRC1	protein regulator of cytokinesis 1	Mitotic spindle assembly	0.97

CDC2	cell division cycle 2	Cell Cycle G1 to S and G2 to M	0.97

CDKN3	cyclin-dependent kinase inhibitor 3	Cell Cycle	0.97

A high AUC score indicates a strong ability to distinguish between groups (in this case, to identify expressors of our genes of interest).

### 3. MAPS is associated with poor prognosis in lung adenocarcinoma

**Figure 4:**
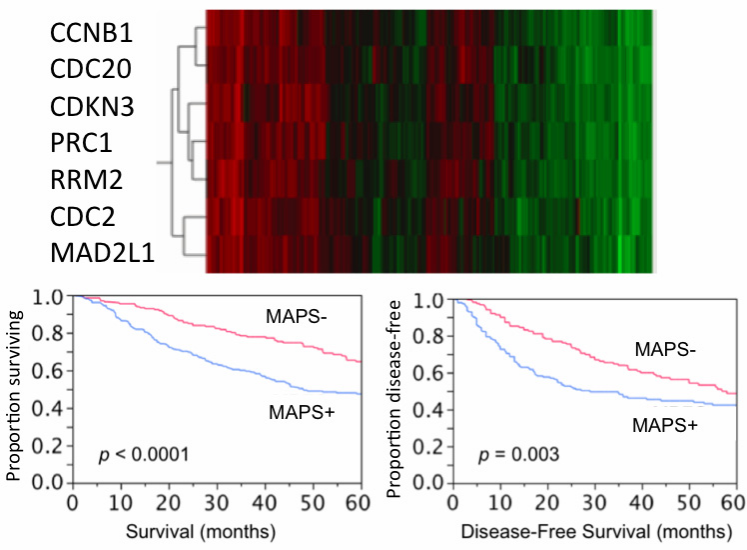
**Expression of genes in the MUC1-Associated Proliferation Signature (MAPS)**. Survival and disease-free survival for expressors of the signature (MAPS+, n = 212) compared with non-expressors (MAPS-, n = 229).

Induction of the MAPS by MUC1-CD and its association with cell proliferation suggested that expression of this signature might identify an aggressive tumor phenotype. To test this hypothesis, we investigated two independent expressional databases of lung adenocarcinomas (see Materials and Methods). Expression of the MAPS in 442 lung adenocarcinomas [23] demonstrated distinct differences in expression across patients (Figure 4). Importantly, patients expressing the MAPS (MAPS+) had a significantly worse prognosis (5-year survival 45% vs. 65%, log-rank *p *< 0.0001, 5-year disease free survival 41% vs. 49%, log-rank *p *= 0.003, Figure 4) compared to non-expressors (MAPS-).

**Figure 5:**
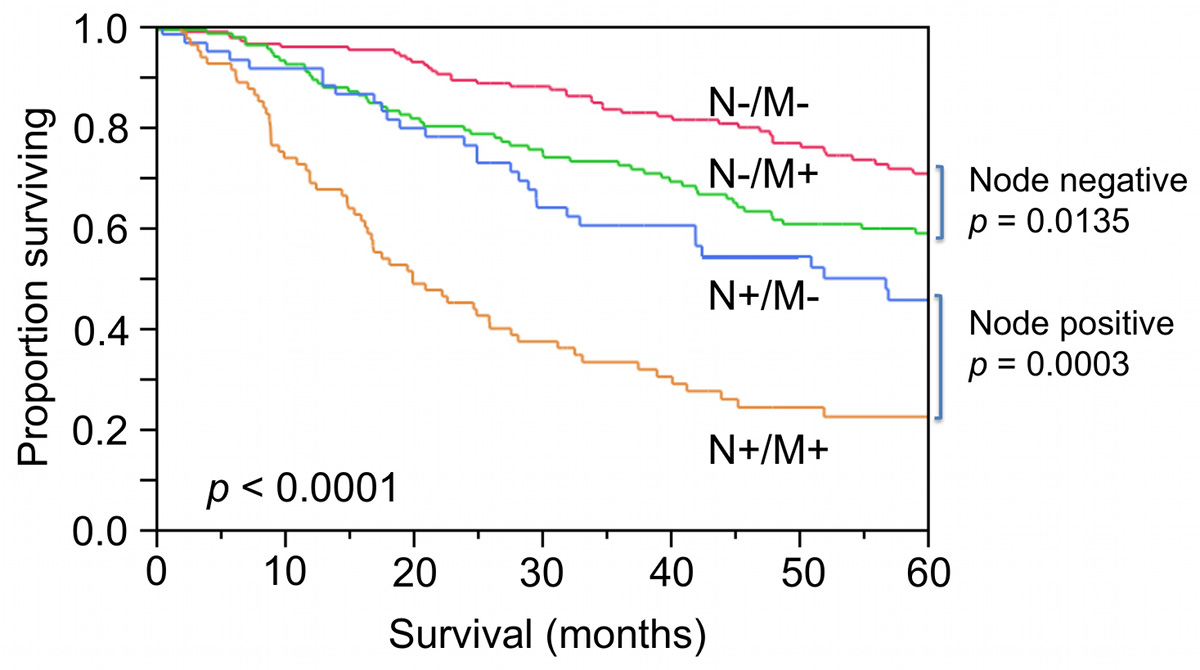
**Survival in all patients in the database in whom lymph node status is known**. Survival is inferior in node-positive patients and in patients who are expressors (MAPS+). MAPS adds significantly to prognostication in both node-negative and node-positive patients.

**Figure 6:**
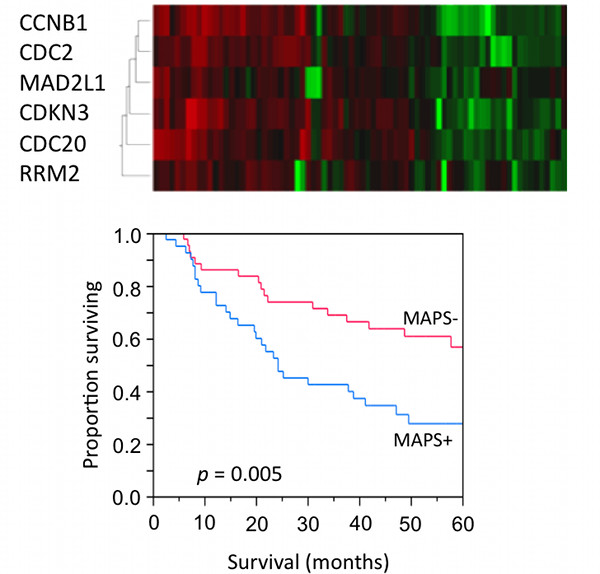
**Expressional clustering of genes in the MAPS signature in a second lung adenocarcinoma database**. Survival for these groups is shown in a Kaplan-Meier curve with expressors (MAPS+, n = 52) and non-expressors (MAPS-, n = 32).

To evaluate the prognostic value of the MAPS, a multivariate Cox proportional analysis, including tumor stage, tumor grade, lymph node involvement and MAPS expression, was performed as described in the Materials and Methods. Distribution of clinical characteristics in the study population is shown in Table 3. The data presented in Table 4 demonstrate that expression of the MAPS is an independent prognostic factor for overall survival (HR = 1.6, *p *= 0.024). Lymph node involvement was the most significant prognostic factor for overall survival based on univariate and multivariate analysis (multivariate HR 2.6, *p *= 2.29 × 10^-11^). Furthermore, expression of the MAPS enhanced the prognostic ability of lymph node status. In this regard, the 5-year survival for MAPS-/node-negative was significantly better than MAPS+/node-negative (71% vs. 59%, *p *= 0.0135) and survival for MAPS-/node-positive was significantly better than MAPS+/node-positive (46% vs. 22%, *p *= 0.0003) (Figure 5). Using an independent database of 84 patients [25] for validation of these results, the data show that patients stratified by expression of these genes also showed significant differences in overall survival (5-year survival 57% in MAPS- vs. 28% in MAPS+, log-rank *p *= 0.005, Figure 6). These data thus support that expression of the genes comprising the MAPS has clinical relevance in the identification of lung adenocarcinoma patients with poor prognosis.

**Table 3:** Clinical variables in 441 lung adenocarcinoma patients included in survival analysis.

Clinical variable	Categories	Number of patients
T stage	T1-2	399
	
	T3-4	40

N stage	Nodes uninvolved (N0)	299
	
	Nodes involved (N1-2)	139

Tumor grade	Low grade (1)	60
	
	Intermediate or high grade (2-3)	374

Our classifier (MAPS)	MAPS-	229
	
	MAPS+ (Expressor)	211

**Table 4:** Univariate and multivariate analysis of clinical variables affecting 5-year survival of lung cancer patients, including our classifier, with hazard ratios (HR).

Clinical variable	Comparison	p-value (univariate)	p-value (multivariate)	HR
T stage	T1-2 vs. T3-4	**1 × 10**^**-7**^	**1.77 × 10**^**-5**^	**2.6**

N stage	Nodes uninvolved (N0) vs. Nodes involved (N1-2)	**2.84 × 10**^**-12**^	**2.29 × 10**^**-11**^	**2.8**

Tumor grade	Low grade (1) vs. intermediate or high grade (2-3)	0.12	0.37	1.3

Our classifier (MAPS)	MAPS- vs. MAPS+ (Expressor)	**4.66 × 10**^**-6**^	**0.0001**	**1.8**

Statistically significant results are bolded.

## Discussion

Previously we found that the cytoplasmic domain of MUC1 (MUC1-CD) induces multiple, stable transcriptional changes in transfected cells. We investigated these changes in *in vivo *models and identified two unique MUC1-CD-dependent expressional signatures. One, which we denoted as the MTS (MUC1 Tumorigenesis Signature), was identified *in vivo *and reflected the interactions of tumor cells with the host microenvironment, as was evidenced by the activation of genes involved in angiogenesis and extracellular matrix signaling [18]. A second expressional signature representing lipid and cholesterol metabolism was also identified in the context of tumor-stromal interactions [17]. Therefore, in the current report, we focused on the detection of MUC1-CD-dependent transcriptional changes unique to oncogenic cells. We hypothesized that these "intrinsic" changes would be important for MUC1-CD signaling leading to oncogenesis and may be connected with the fundamental mechanisms of the malignant transformation. In this regard, we profiled MUC1-CD-transformed cells grown *in vitro*, without any influences from the host stroma.

In the current report, we describe a MUC1-Associated Proliferation Signature (MAPS) that provides independent prognostic information, adding to standard pathological evaluation and clinical staging of lung adenocarcinoma. This signature was derived from the set of genes initially detected in an experimental system as upregulated by MUC1-CD *in vitro *and potentially involved in a highly oncogenic phenotype [18]. The MAPS is distinct from our previously reported MUC1-related signature (MTS, [18]) and was identified using a contrasting experimental approach. The major functional groups of differentially expressed genes *in vitro *represented cellular growth, proliferation and cell cycle control. In comparison, the MTS was derived from genes highly enriched by functional groups representing cell motility, metastasis and angiogenesis. We believe that these results demonstrate important differences between the intrinsic properties of tumor cells and the properties that are determined by tumor-stromal interactions. Interestingly, only two genes (CDC20 and RRM2) were common between both signatures, perhaps indicating that, at least in our experimental system, expression of these two genes is independent of the host microenvironment.

All the genes that comprise the MAPS are related to cell cycle control and proliferation. For instance, CDC20 (homolog of S. cerevisiae cell division cycle 20 protein) directly binds to and activates anaphase-promoting complex (APC), which leads to ubiquitination and degradation of cyclin B (CCNB1) and therefore promotes the onset of anaphase and mitotic exit [28]. The APC/CDC20 complex is under negative control of MAD2L1 (human homolog of S. Cerevisiae MAD2) and BUB1 (see Table 1). Also, PRC1 (protein regulating cytokinesis 1) is a human homolog of S. cerevisiae Ase1, which is involved in spindle formation and also promotes anaphase and mitotic exit [29]. CDC2 (cell division cycle 2), or CDK1 (cyclin-dependent kinase 1), is a catalytic subunit of a protein kinase complex, called the M-phase promoting factor, formed with cyclin B1 (CCNB1) that induces entry into mitosis [30]. CDC2 phosphorylates securin, which is another target of APC/CDC20 and is an inhibitor of separase-protease, responsible for the cleavage of sister chromatid cohesions. CDC2-dependent phosphorylation of securin protects it from APC/CDC20-induced ubiquitination and degradation [31]. CDKN3 (cyclin-dependent kinase inhibitor 3) is a dual-specificity protein phosphatase that interacts with CDC2 and CDK2 and inhibits their activity [32]. These data show that six of the seven genes comprising MAPS not only belong to a cell cycle-related functional group but represent a specific pathway of interacting proteins responsible for anaphase control, chromosome segregation and mitotic entrance/exit (see also Figure 1B). RRM2 (ribonucleotide reductase, M2 subunit) encodes the small subunit (R2) of ribonucleotide reductase, the heterodimeric enzyme that catalyzes the rate-limiting step in deoxyribonucleotide synthesis. Using siRNA screening, Kittler et al. [33] identified 37 genes required for cell division, one of which was RRM2.

There is substantial literature indicating that the genes in MAPS are co-expressed and are involved in tumorigenesis and cancer progression. Five of seven MAPS genes are upregulated in immortalized breast cancer cell lines compared to primary breast tumor cell cultures (CDC2, CDC20, CDKN3, MAD2L1 and RRM2) [34] and all seven MAPS genes are upregulated in response to infection of HPV-18, a virus associated with cervical cancer, in keratinocytes [35]. All seven were also found to be co-expressed with E2F, which is expressed in breast cancer compared with normal breast tissue and is elevated during the G2/M transition [36]. This suggests a possible role for E2F inhibitors in treating poor-prognosis cancers that express MAPS. All seven MAPS genes are downregulated in response to *Brd4 *transfection in a mouse mammary cell line and are included in a 141-gene prognostic signature based on differential expression in this cell line. Expression of this signature correlated with prognosis in five separate human breast cancer cohorts [37]. This is one of many published results from tumor expression profiling experiments which have linked increased expression of genes from common pathways involved in cell growth and proliferation to poor outcomes in cancer patients [38]. A meta-signature was identified consisting of sixty-nine genes expressed more in high-grade compared to low-grade tumors in eight separate microarray analyses spanning seven types of cancer including lung adenocarcinoma [39]. These included many genes associated with cell proliferation, including five of the seven genes in our abbreviated MAPS signature: CCNB1, CDC2, CDC20, CDKN3 and MAD2L1. Thus, MAPS reflects a pattern of gene expression associated with high-grade cancers, but having greater prognostic significance than histological grade in our results.

Current data of Whitfield et al. [38] indicate that proliferation-associated genes can be considered not only as common prognostic/predictive markers in different cancers, but also as promising targets for anti-cancer therapy. Among the genes comprising MAPS, at least two are targets of known drugs. These are hydroxyurea for RRM2 and flavopiridol and staurosporin (UCN-01) for CDK1 (CDC2). In addition, taxol, which affects microtubule formation and blocks mitosis at the G2/M transition, may have interactions with 6 of 7 gene products included in MAPS. RRM2 is also a target that may be used to potentiate chemotherapy. Kittler et al. [33] demonstrated that silencing of RRM1 and RRM2, which encode the large and small subunits of the human ribonucleotide reductase (RNR) complex, respectively, markedly enhanced the cytotoxicity of the topoisomerase I (Top1) inhibitor camptothecin (CPT). Silencing of RRM2 was also found to enhance DNA damage as measured by γ-H2AX. Upregulation in RRM2 expression levels suggests an active role for RNR in the cellular response to DNA damage that could potentially be exploited as strategy for enhancing the efficacy of Top1 inhibitors [40]. The MUC1-CD is also involved in the control of DNA damage response [41]. The data presented in the current report suggest that this control may be associated with a set of genes regulating G2/M transition and exit from mitosis through the network of reactions connected with spindle formation and chromosome segregation.

Many existing biomarkers that have been identified for non-small-cell lung cancer indicate the presence of disease, as in screening or recurrence. The genes in the MAPS are, however, potential biomarkers of prognosis and could help guide treatment in patients with a new diagnosis of primary lung cancer. There are cytokeratin biomarkers that have been studied which show evidence of prognostic significance in lung adenocarcinoma, including CYFRA 21-1, TPA and TPS [42]. These biomarkers are detected on the protein levels in relatively high concentrations from freshly prepared tissues. Our signature has the potential to be measured by PCR at picogram levels both in frozen tissues and paraffin-embedded archival samples. Further prospective investigations are needed to compare potential protein and RNA-based biomarkers, which might be complementary to each other.

## Conclusions

These data indicate new insights into the mechanisms through which MUC1-CD performs its DNA damage-response and tumorigenic functions. They also suggest targets that can be accessed for tumor suppression/sensitization to genotoxic treatments in a MUC1-CD-dependent pathway. Therefore, the MUC1-Associated Proliferation Signature (MAPS) described in the current report not only serves as a new classifier, but also sheds light on the mechanisms of MUC1-CD-associated tumorigenesis and suggests potential gene products and drugs for targeted cancer therapy.

## Competing interests

D. Kufe holds equity in Genus Oncology and is a consultant to the company.

## Authors' contributions

DMM and NNK designed the study, carried out data analysis and drafted the manuscript. SPP participated in data analysis and helped draft the manuscript. DCE carried out ROC analysis and generated ROC curves. CAP participated in design and coordination of the ROC analysis. LH helped with data collection and analysis. DWK and RRW coordinated the entire study, designed the concept and helped draft the manuscript. All authors read and approved the final manuscript.

## Pre-publication history

The pre-publication history for this paper can be accessed here:

http://www.biomedcentral.com/1755-8794/3/16/prepub

## Supplementary Material

Additional File 1 **Figure S1**. Algorithm used to select a short signature from our biologically derived set of genes correlated with MUC1 transfection. Click here for file

Additional File 2 **Table S1**. 254 genes differentially expressed in MUC1 transfected cells. Click here for file

Additional File 3 **Table S2**. The top two functional networks represented by 254 genes with expressional changes associated with MUC1 transfection. Click here for file

Additional File 4 **Figure S2**. The top functional network represented by 42 selected genes with expressional changes associated with MUC1 transfection and prognostic significance in lung adenocarcinoma patients. Click here for file
