# Supplementary material for: MUC1-associated proliferation signature predicts outcomes in lung adenocarcinoma patients
Source: BMC Med Genomics. 2010 May 6;3:16. doi: 10.1186/1755-8794-3-16 (PMC2876055; doi:10.1186/1755-8794-3-16)

**Figure S1.** Algorithm used to select a short signature from our biologically derived set of genes correlated with MUC1 transfection.


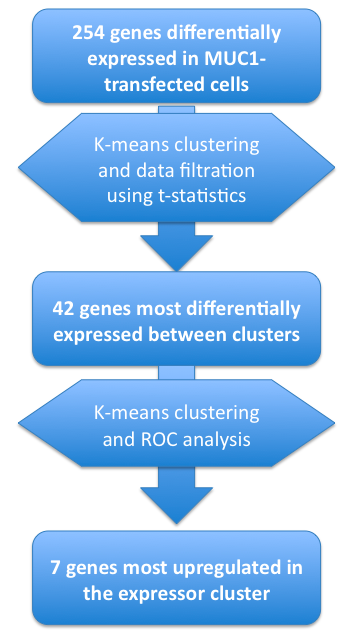

Supplement: Additional File 1 — Figure S1. Algorithm used to select a short signature from our biologically derived set of genes correlated with MUC1 transfection. [file 1755-8794-3-16-S1.DOC]
