# Supplementary material for: MUC1-associated proliferation signature predicts outcomes in lung adenocarcinoma patients
Source: BMC Med Genomics. 2010 May 6;3:16. doi: 10.1186/1755-8794-3-16 (PMC2876055; doi:10.1186/1755-8794-3-16)
Supplement: Additional File 2 — Table S1. 254 genes differentially expressed in MUC1 transfected cells. [file 1755-8794-3-16-S2.DOC]

**Table S1.** 254 genes differentially expressed in MUC1 transfected cells (relative underexpression in left column, relative overexpression in right column).

| **Gene Symbol** | **Relative Underexpression** | **Gene Symbol** | **Relative Overexpression** |
| --- | --- | --- | --- |
| EID2 | 0.010 | TCEAL1 | 1171.409 |
| TGFBI | 0.012 | SNCG | 442.179 |
| COL11A1 | 0.013 | ADH1C | 395.910 |
| HEPH | 0.019 | GDA | 301.558 |
| CRYAB | 0.025 | RIPK4 | 273.936 |
| PTN | 0.028 | PCDH9 | 236.711 |
| OGN | 0.035 | LOC680227 | 221.026 |
| LRRC17 | 0.041 | MMP10 | 167.050 |
| JDP2 | 0.049 | STEAP4 | 166.155 |
| IFI30 | 0.058 | CBLN2 | 160.324 |
| SLC24A3 | 0.063 | CTSC | 153.519 |
| KAZALD1 | 0.086 | RYR3 | 104.098 |
| KLF2 | 0.095 | SERPINB2 | 100.793 |
| C4ORF18 | 0.096 | MMP3 | 100.217 |
| C9ORF150 | 0.106 | AEBP1 | 92.597 |
| MT1E | 0.111 | GPR176 | 88.009 |
| FAM151B | 0.122 | SCIN | 79.104 |
| SULF2 | 0.153 | PTGS1 | 67.029 |
| CSRP2 | 0.153 | SFRP4 | 56.252 |
| METTL7A | 0.159 | PROCR | 51.505 |
| ZFPM2 | 0.161 | HS3ST1 | 50.940 |
| EMB | 0.164 | ENPP3 | 34.452 |
| TMED3 | 0.167 | AREG | 33.625 |
| CALCOCO1 | 0.171 | CD38 | 28.806 |
| CTNNAL1 | 0.181 | SLC4A7 | 27.420 |
| SLC25A36 | 0.186 | MANBA | 25.688 |
| CDC42EP3 | 0.187 | COL8A1 | 24.886 |
| SOX4 | 0.188 | C2ORF64 | 22.547 |
| LOC301977 | 0.199 | IL1RL1 | 21.652 |
| FAM116A | 0.209 | PLCD4 | 21.382 |
| AP4B1 | 0.213 | LAMA4 | 17.859 |
| ALDH6A1 | 0.219 | SLC17A6 | 17.358 |
| ID1 | 0.221 | MMAA | 15.796 |
| KDELR3 | 0.222 | EIF3C | 15.321 |
| CASC4 | 0.231 | SORBS2 | 15.033 |
| METTL6 | 0.246 | XRN2 | 13.940 |
| RPS24 | 0.247 | RGS17 | 12.301 |
| PGCP | 0.251 | GCHFR | 11.947 |
| NUDT4 | 0.256 | HMGA1 | 10.426 |
| HEXB | 0.260 | TMEM16A | 9.553 |
| GNL3 | 0.263 | PTGS2 | 9.492 |
| ARF3 | 0.263 | SASS6 | 8.975 |
| RP51000-E10.4 | 0.269 | PPL | 8.659 |
| COL1A1 | 0.270 | PCDH19 | 8.630 |
| PUF60 | 0.270 | ABCG1 | 8.565 |
| FN3KRP | 0.279 | SLC29A1 | 8.423 |
| VKORC1 | 0.280 | IFI16 | 8.205 |
| GOLIM4 | 0.282 | SSX2IP | 8.164 |
| AASS | 0.284 | MST150 | 7.464 |
| ZBTB1 | 0.285 | PTPRF | 7.335 |
| ATF4 | 0.286 | USP33 | 7.271 |
| NDRG1 | 0.294 | DHCR24 | 7.258 |
| LEMD3 | 0.304 | PCDH18 | 7.018 |
| NUDT4 | 0.304 | LPAR1 | 6.522 |
| SORD | 0.309 | GARNL4 | 6.241 |
| RGD1310591 | 0.313 | DUSP6 | 6.194 |
| RGD1566118 | 0.316 | PVR | 6.123 |
| OLFML3 | 0.318 | TMED5 | 5.843 |
| SIX1 | 0.319 | C15ORF48 | 5.728 |
| TOMM22 | 0.320 | CYP51A1 | 5.708 |
| CDKN1A | 0.322 | PCDH7 | 5.706 |
| FBLN5 | 0.323 | CD14 | 5.560 |
| COPZ2 | 0.326 | ACAT2 | 5.481 |
| PTGFRN | 0.327 | DIAPH3 | 5.339 |
| CHD1L | 0.330 | EHD4 | 5.227 |
| LOC688318 | 0.334 | CTNND2 | 5.226 |
| C12ORF62 | 0.339 | DAG1 | 5.148 |
| CCDC80 | 0.342 | DUSP5 | 5.050 |
| RNASET2 | 0.342 | F3 | 5.029 |
| GPNMB | 0.344 | LMO7 | 4.979 |
| JOSD1 | 0.349 | CTH | 4.842 |
| KIAA1737 | 0.350 | MAD2L1 | 4.811 |
| INTS10 | 0.350 | LPCAT3 | 4.787 |
| HIPK1 | 0.352 | STARD13 | 4.659 |
| ANKRD10 | 0.353 | NSDHL | 4.622 |
| MYBBP1A | 0.354 | CDKN3 | 4.608 |
| CHKB | 0.356 | MMD | 4.603 |
| BCAS2 | 0.356 | ELOVL6 | 4.410 |
| COL3A1 | 0.359 | HEY2 | 4.379 |
| NNT | 0.359 | CKAP4 | 4.350 |
| LRIG3 | 0.363 | PCBP3 | 4.336 |
| MRPS30 | 0.366 | C20ORF191 | 4.328 |
| GHITM | 0.369 | IDI1 | 4.272 |
| NAP1L1 | 0.369 | CTBS | 4.269 |
| SDC2 | 0.370 | ERCC1 | 4.216 |
| ANKRD28 | 0.377 | UBXD2 | 4.186 |
| ZC3H7B | 0.378 | GREM1 | 4.176 |
| UNC84B | 0.381 | NID1 | 4.171 |
| PSAT1 | 0.382 | METAP1 | 4.137 |
| WBP2 | 0.386 | CSNK2A1 | 4.073 |
| ATXN10 | 0.389 | ATG12 | 4.067 |
| CPSF1 | 0.398 | AKAP7 | 3.997 |
| HCG2023776 | 0.399 | ETV4 | 3.935 |
| COPE | 0.401 | BCL10 | 3.822 |
| APEX1 | 0.406 | DEPDC1 | 3.812 |
| CPSF6 | 0.406 | DNMBP | 3.806 |
| FRS2 | 0.409 | SFRS11 | 3.805 |
| ERN1 | 0.414 | TMEM87B | 3.750 |
| EIF3H | 0.420 | RAB32 | 3.725 |
| TBL1XR1 | 0.424 | LPHN2 | 3.723 |
| ITM2B | 0.428 | USP18 | 3.663 |
| EEF1D | 0.429 | MBOAT1 | 3.650 |
| MRPS7 | 0.430 | HECTD1 | 3.645 |
| ARL6IP2 | 0.443 | SLC20A1 | 3.637 |
| SLC16A1 | 0.446 | LOC678766 | 3.535 |
| FUCA1 | 0.448 | FDPS | 3.489 |
| TSNAX | 0.449 | CCL13 | 3.430 |
| PPM1L | 0.452 | RPL27A | 3.397 |
| COX6A1 | 0.454 | PKN2 | 3.373 |
| NOL1 | 0.457 | PFDN1 | 3.371 |
| TIMP2 | 0.458 | KLHL30 | 3.369 |
| SRP72 | 0.460 | RALA | 3.347 |
| CXCL10 | 0.463 | MMP13 | 3.326 |
| MCM7 | 0.473 | SF3B1 | 3.312 |
| GRN | 0.475 | GPRC5B | 3.292 |
| TTC35 | 0.479 | CLDND1 | 3.275 |
| PRR13 | 0.480 | PTTG1 | 3.195 |
| PPP2R5E | 0.485 | DHCR7 | 3.185 |
| CCNG2 | 0.486 | EIF2C2 | 3.130 |
| WDR68 | 0.487 | GTF2B | 3.103 |
| TRIM35 | 0.487 | CUGBP1 | 3.099 |
| FAM91A1 | 0.488 | TRIM25 | 3.055 |
| SLC30A5 | 0.488 | OLFM1 | 3.053 |
| SCHIP1 | 0.489 | PJA2 | 3.048 |
| TSC22D1 | 0.491 | CCNB2 | 3.047 |
| BECN1 | 0.493 | PON2 | 3.002 |
| C12ORF41 | 0.496 | SPRY2 | 2.935 |
|  |  | EDEM1 | 2.911 |
|  |  | SH2B3 | 2.895 |
|  |  | PHPT1 | 2.863 |
|  |  | LRRFIP1 | 2.807 |
|  |  | ANLN | 2.777 |
|  |  | TRIM8 | 2.761 |
|  |  | HS2ST1 | 2.751 |
|  |  | PRC1 | 2.743 |
|  |  | LOC687090 | 2.740 |
|  |  | ADAM17 | 2.730 |
|  |  | TCAG7.1260 | 2.711 |
|  |  | C1R | 2.700 |
|  |  | ALCAM | 2.648 |
|  |  | DNAJC9 | 2.627 |
|  |  | PLAT | 2.594 |
|  |  | CDC2 | 2.591 |
|  |  | FADS1 | 2.591 |
|  |  | CPD | 2.580 |
|  |  | CTTN | 2.569 |
|  |  | CLIC1 | 2.534 |
|  |  | BTG3 | 2.511 |
|  |  | SH3BGRL3 | 2.448 |
|  |  | PLSCR1 | 2.398 |
|  |  | HPS6 | 2.390 |
|  |  | CLCN3 | 2.386 |
|  |  | CDC20 | 2.374 |
|  |  | TCF19 | 2.370 |
|  |  | CD44 | 2.347 |
|  |  | BUB1 | 2.342 |
|  |  | SLC25A24 | 2.332 |
|  |  | TXNDC13 | 2.327 |
|  |  | ARL4A | 2.318 |
|  |  | ESM1 | 2.309 |
|  |  | EZR | 2.243 |
|  |  | SLK | 2.235 |
|  |  | KIF22 | 2.229 |
|  |  | SC5DL | 2.225 |
|  |  | MAPK6 | 2.204 |
|  |  | PMVK | 2.190 |
|  |  | PRKACB | 2.144 |
|  |  | TES | 2.121 |
|  |  | NTAN1 | 2.103 |
|  |  | H2AFZ | 2.084 |
|  |  | RBM5 | 2.082 |
|  |  | ADFP | 2.081 |
|  |  | CCNB1 | 2.080 |
|  |  | RRM2 | 2.064 |
|  |  | PFN2 | 2.042 |
|  |  | FKBP2 | 2.036 |
|  |  | ABI1 | 2.025 |
|  |  | LOC682999 | 2.024 |
|  |  | ACSL3 | 2.013 |
