# Supplementary material for: MUC1-associated proliferation signature predicts outcomes in lung adenocarcinoma patients
Source: BMC Med Genomics. 2010 May 6;3:16. doi: 10.1186/1755-8794-3-16 (PMC2876055; doi:10.1186/1755-8794-3-16)
Supplement: Additional File 3 — Table S2. The top two functional networks represented by 254 genes with expressional changes associated with MUC1 transfection. [file 1755-8794-3-16-S3.DOC]

**Table S2.** The top two functional networks represented by 254 genes with expressional changes associated with MUC1 transfection.

| **Functions within Network** | **Molecules from 254-gene set present in network** | **Additional molecules in network (not in 254-gene set)** |
| --- | --- | --- |
| Cellular Growth and Proliferation, Inflammatory Disease, Cellular Movement | ABCG1, AEBP1, ATF7, BCL10, C1R, C1S, CCL13, CD14, CD38, CD44, CKAP4, CSNK2A1, CXCL10, DUSP5, F3, FUCA1, GTF2B, HMGA1, IL1RL1, MMP3, MMP13, PLAT, PON2, PVR, RIPK4, SERPINB2, TIMP2 | AP1, CD3, IL1, LDL, MMP, NFkB, TGF beta, VitaminD3-VDR-RXR |
| Cell Cycle, Cellular Assembly and Organization, DNA Replication, Recombination, and Repair | Alkaline Phosphatase, ATF4, BUB1, CCNB1, CCNB2, CDC2, CDC20, CDKN3, CDKN1A, CRYAB, CTH, CUGBP1, H2AFZ, ID1, IFI16, KLF2, MAD2L1, MCM7, NDRG1, PRC1, PSAT1, PTTG1, RRM2, SDC2, SF3B1, SLC20A1, SLC30A5 | Cyclin A, Cyclin B, Cyclin E, E2f, Insulin, Mek1/2, Rb, RNA polymerase II |
