# Supplementary material for: MUC1-associated proliferation signature predicts outcomes in lung adenocarcinoma patients
Source: BMC Med Genomics. 2010 May 6;3:16. doi: 10.1186/1755-8794-3-16 (PMC2876055; doi:10.1186/1755-8794-3-16)

**Figure S2.** The top functional network represented by 42 selected genes with expressional changes associated with MUC1 transfection and prognostic significance in lung adenocarcinoma patients.

| **Functions within Network** | **Molecules in 42-gene set present in network** | **Additional molecules in network (not in the 42-gene set)** |
| --- | --- | --- |
| DNA Replication, Recombination, and Repair, Cell Cycle, Cancer | BECN1, BUB1, CCNB1, CDC2, CDC20, CDKN3, CKAP4, CSRP2, DHCR24, DUSP6, ERN1, ETV4, FRS2, FUCA1, ID1, MAD2L1, MCM7, PFN2, PTPRF, RYR3, SLC20A1 | BIC, Ccnb1-Cdc2, Cdc2-CyclinB, Cyclin A, Cyclin E, ERK, FSH, Jnk, MAP2K1/2, Mapk, NFkB, P38 MAPK8, PDGF BB, RNA polymerase II |


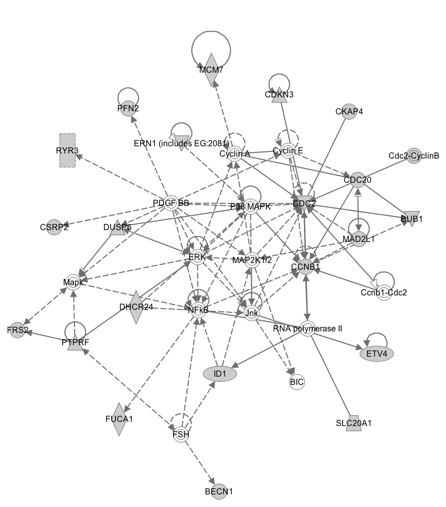

Supplement: Additional File 4 — Figure S2. The top functional network represented by 42 selected genes with expressional changes associated with MUC1 transfection and prognostic significance in lung adenocarcinoma patients. [file 1755-8794-3-16-S4.DOC]
